# Supplementary material for: Exploring children and young people’s experience of participating in citizen science–A qualitative evidence synthesis
Source: PLoS One. 2024 Jul 11;19(7):e0304976. doi: 10.1371/journal.pone.0304976 (PMC11239061; doi:10.1371/journal.pone.0304976)
Supplement: S1 Table — (DOCX) [file pone.0304976.s002.docx]

## S1. Enhancing transparency in reporting the synthesis of qualitative research: ENTREQ Checklist

| Item No. | Guide and Description | Description and Report Location |
| --- | --- | --- |
| 1. Aim | State the research question the synthesis addresses | Aims and Research Objectives P6 |
| 2. Synthesis methodology | Identify the synthesis methodology or theoretical framework which underpins the synthesis, and describe the rationale for choice of methodology (e.g. meta-ethnography, thematic synthesis, critical interpretive synthesis, grounded theory synthesis, realist synthesis, meta-aggregation, meta-study, framework synthesis) | Methods - Thematic synthesis p 9 |
| 3. Approach to searching | Indicate whether the search was pre-planned (comprehensive search strategies to seek all available studies) or iterative (to seek all available concepts until they theoretical saturation is achieved) | Methods – Literature search p7-9 |
| 4. Inclusion criteria | Specify the inclusion/exclusion criteria (e.g. in terms of population, language, year limits, type of publication, study type) | Methods – Inclusion and exclusion criteria P7-8 |
| 5. Data sources | Describe the information sources used (e.g. electronic databases (MEDLINE, EMBASE, CINAHL, psycINFO), grey literature databases (digital thesis, policy reports), relevant organisational websites, experts, information specialists, generic web searches (Google Scholar) hand searching, reference lists) and when the searches conducted; provide the rationale for using the data sources | Methods – Literature search P7-8  Figure 1 - Search flow chart |
| 6. Electronic Search strategy | Describe the literature search (e.g. provide electronic search strategies with population terms, clinical or health topic terms, experiential or social phenomena related terms, filters for qualitative research, and search limits) | Methods – Literature Search p 7 and Supplementary material 2 – key search terms |
| 7. Study screening methods | Describe the process of study screening and sifting (e.g. title, abstract and full text review, number of independent reviewers who screened studies) | Methods- Thematic Synthesis P 9 |
| 8. Study characteristics | Present the characteristics of the included studies (e.g. year of publication, country, population, number of participants, data collection, methodology, analysis, research questions) | Supplementary Material 3 and 4 |
| 9. Study selection results | Identify the number of studies screened and provide reasons for study exclusion (e.g. for comprehensive searching, provide numbers of studies screened and reasons for exclusion indicated in a figure/flowchart; for iterative searching describe reasons for study exclusion and inclusion based on modifications to the research question and/or contribution to theory development) | Methods – selecting the literature p9  Figure 1 - Search flow chart |
| 10. Rationale for appraisal | Describe the rationale and approach used to appraise the included studies or selected findings (e.g. assessment of conduct (validity and robustness), assessment of reporting (transparency), assessment of content and utility of the findings) | Methods – Quality assessment P11  Supplementary material 5 |
| 11. Appraisal items | State the tools, frameworks and criteria used to appraise the studies or selected findings (e.g. Existing tools: CASP, QARI, COREQ, Mays and Pope [25]; reviewer developed tools; describe the domains assessed: research team, study design, data analysis and interpretations, reporting) | Methods – Quality assessment P11  Supplementary material 4 |
| 12. Appraisal process | Indicate whether the appraisal was conducted independently by more than one reviewer and if consensus was required | Methods – Quality assessment p 9 |
| 13. Appraisal results | Present results of the quality assessment and indicate which articles, if any, were weighted/excluded based on the assessment and give the rationale | Methods- Quality Assessment p911  Supplementary material 4 |
| 14. Data extraction | Indicate which sections of the primary studies were analysed and how were the data extracted from the primary studies? (e.g. all text under the headings “results /conclusions” were extracted electronically and entered into a computer software) | Selecting literature p9  Method- Thematic synthesis p13 |
| 15. Software | State the computer software used, if any | Methods Literature Search p7  Thematic Synthesis p9 |
| 16. Number of reviewers | Identify who was involved in coding and analysis | Methods – Thematic Synthesis p9. Author contribution (end of manuscript) |
| 17. Coding | Describe the process for coding of data (e.g. line by line coding to search for concepts) | Methods – Thematic Synthesis p 9 |
| 18. Study comparison | Describe how were comparisons made within and across studies (e.g. subsequent studies were coded into pre-existing concepts, and new concepts were created when deemed necessary) | Methods – Thematic Synthesis p 9 |
| 19. Derivation of themes | Explain whether the process of deriving the themes or constructs was inductive or deductive | Methods – Thematic Synthesis p 9 |
| 20. Quotations | Provide quotations from the primary studies to illustrate themes/constructs, and identify whether the quotations were participant quotations of the author’s interpretation | Results – Thematic Synthesis p14-31 |
| 21. Synthesis output | Present rich, compelling and useful results that go beyond a summary of the primary studies (e.g. new interpretation, models of evidence, conceptual models, analytical framework, development of a new theory or construct) | Figure 1 The themes and aspects relating to participant experience p 11  Discussion p31-38 |
