# Supplementary material for: Exploring children and young people’s experience of participating in citizen science–A qualitative evidence synthesis
Source: PLoS One. 2024 Jul 11;19(7):e0304976. doi: 10.1371/journal.pone.0304976 (PMC11239061; doi:10.1371/journal.pone.0304976)
Supplement: Attachment — Submitted filename: response.docx [file pone.0304976.s007.docx]

Response

A previous submission (PONE-D-23-11833) was rejected as the synthesis conducted on Jan 2022 did not provide an up-to-date overview of the research topic covered.

We were advised by the editor Jaskirat Singh that a revised, updated manuscript could be considered.

This manuscript contains an update to the synthesis conducted in May 2023.

Using the same search strategy a systematic review of the literature was carried out to include literature up to 5^th^ May 2023.

The manuscript was reworked to include the update to the synthesis across all sections.

We deeply appreciate the valuable feedback provided on our original submission and we are delighted to share the improvements we have made in response.

The updated synthesis not only added depth to our original themes but also highlighted an increase in papers focusing young people experiences of taking part in citizen science projects. This discovery further underscores the timeliness and relevance of our synthesis.
