# Supplementary material for: Exploring children and young people’s experience of participating in citizen science–A qualitative evidence synthesis
Source: PLoS One. 2024 Jul 11;19(7):e0304976. doi: 10.1371/journal.pone.0304976 (PMC11239061; doi:10.1371/journal.pone.0304976)
Supplement: Attachment — Submitted filename: Response to Reviewers.docx [file pone.0304976.s008.docx]

Dear Editor,

We appreciate the time and care you took to find suitable reviewers. We have revisited the PLOSone guidelines and made the changes suggested as detailed below.

Please can we extend our thanks to the reviewers. Thank you both for your thorough and thoughtful reviews of the synthesis. We were grateful for the positive remarks and feel we have made changes to the paper that have improved the quality and content. Additionally, you have opened up new areas of focus for future research and provided us with valuable insights.

Reviewer 1, Thank you for your kind words and identifying the need for the paper in your review, we are passionate about young people in citizen science and to have the need for this paper recognised was uplifting. We are proud that our thorough search and update were noted. We greatly appreciate the time you have taken to suggest improvements. We enjoyed reading the suggested papers, particularly those related to citizen social science and stakeholders involved in citizen science. These recommendations have not only helped improve this paper but we will continue to use the work discussed and ideas proposed in future work, we are grateful you took the time to include these papers. The revisions you have suggested have been addressed below.

Reviewer 2, Thank you for acknowledging the work that updated the synthesis, and supporting the need for the paper. We appreciate the time you have taken to diligently read through the paper and make your useful comments. Your suggestions have helped improve the flow of the paper. We have also specifically revisited the communication theme to strengthen this section, and the wider impacts theme to clarify what this section is for. The inclusion of a code book we hope will add to the transparency of the work.

**The editors and reviewers’ comments are addressed in full below.**

Please can you advise on the quality of the figures we have uploaded and if they are readable? We have resubmitted figures 1 and 2.

| Comments |  |
| --- | --- |
| Journal requirements |  |
| Please ensure that your manuscript meets PLOS ONE's style requirements, including those for file naming. The PLOS ONE style templates can be found at  <https://journals.plos.org/plosone/s/file?id=wjVg/PLOSOne_formatting_sample_main_body.pdf> and   <https://journals.plos.org/plosone/s/file?id=ba62/PLOSOne_formatting_sample_title_authors_affiliations.pdf> | We have revisited the plosone style requirements and now hope the documents comply with the templates. |
| 2. Please be informed that funding information should not appear in the Acknowledgments section or other areas of your manuscript. We will only publish funding information present in the Funding Statement section of the online submission form. Please remove any funding-related text from the manuscript. | Please accept our apology for misunderstanding the PLOSONE guidelines the funding information has been removed from the manuscript. Page 45 |
| 3. Thank you for stating the following financial disclosure: "The author(s) received no specific funding for this work."  At this time, please address the following queries:  a) Please clarify the sources of funding (financial or material support) for your study. List the grants or organizations that supported your study, including funding received from your institution.   b) State what role the funders took in the study. If the funders had no role in your study, please state: “The funders had no role in study design, data collection and analysis, decision to publish, or preparation of the manuscript.”  c) If any authors received a salary from any of your funders, please state which authors and which funders.  d) If you did not receive any funding for this study, please state: “The authors received no specific funding for this work.”  Please include your amended statements within your cover letter; we will change the online submission form on your behalf. | a) This study is funded by Sport England and the University of Bradford (match funding) via a PhD studentship. Sport England is a non-departmental public body under the Department for Digital, Culture, Media and Sport. The views expressed in this publication are those of the author(s) and not necessarily those of Sport England nor the Department for Digital, Culture, Media and Sport. b) The funders had no role in study design, data collection and analysis, decision to publish, or preparation of the manuscript. c) Authors, A.D-S and J.H. were supported by Sport England’s Local Delivery Pilot—Bradford; weblink: [**https://www.sportengland.org/campaigns-and-our-work/local-delivery**](https://www.sportengland.org/campaigns-and-our-work/local-delivery) (accessed on 24th January 2024). |
| Editor |  |
| Why has the librarian not been included as an author or in the least official acknowledgement by name in the acknowledgement section? o “The research team (MF, JH, ADS, MT) and librarian developed 147 search strategies (S2 Key search terms)” | Thank you for pointing out our oversight. The librarian has been added to the acknowledgements. |
| Please mention the 7 databases in the body of the manuscript – and why you have selected these 7 databases – either providing a reason for each – or an overall reason | The databases have been added to the main text, with an overall justification for why they were chosen. Page 8 |
| Different fonts used in the manuscript – looks like Times New Roman and Arial – | The font has all been standardised to Arial. |
| Duplication of paragraphs – just before the conclusion | Apologies for this oversight, the duplicate paragraph has been removed. |
| Reviewer 1 |  |
| Major |  |
| 1. Reconsider the presentation of work steps by authors in the main text, possibly using CRediT or a separate declaration. | Thank you for this comment, we want our work to be transdisciplinary, and appreciate you drawing our attention to the fact that having the authors in the main work may not be seen as appropriate. I have removed the authors names from the main text and added them into a declaration. In line with the ENTREQ statement ‘Identify who was involved in coding and analysis’ we wanted it to be clear to the reader who had screened, appraised coded and analysed for both the original and updated search we feel this can be achieved in the declaration. |
| 2. Enhance the results section with a clear presentation of factors and their occurrence in the studied articles. | Thank you for this comment, we have included a table (Table 1) to show the number of codes per theme. Additionally, by including our code book there is more clarity on how the themes were created. We see the value in this transparency. We have not included a tally in the main text as taking the inductive approach an element may only have been mentioned once, but precisely because of this uniqueness may have been included and is still a valid element of participant experience. We have not conducted a content or frequency analysis of the factors mentioned and feel this would be a separate study. |
| 3. Provide a breakdown of the factors based on the type of Citizen Science approach. | Thank you for drawing our attention to a different way of viewing the data. We have added Table 1 which shows the number of codes per theme according to different categories of citizen science (Page 13). In Supplementary table 3 we have the type of citizen science noted. It is important to see that experiences do differ according to research methodology. As a group we had discussed this as a way to approach the synthesis, however, we wanted to look at young people’s experiences overall across projects and the key themes. We see young people’s experiences in research as vital and would like to complete future research into how the experiences differ across different disciplines and types of citizen science but believe there is not scope to fully achieve this in this synthesis. We have added this comment to the future suggestion section page 45 |
| 4. Delve deeper into the nature of the CS-projects to ascertain the balance between natural/technical and humanities/social sciences. | Thank you for sharing how the data can be looked at from various viewpoints, and the importance of the research discipline. The type of project has been added to Summary table 3. Within the text, citizen science and the disciples it is associated with are now discussed in the introduction section (page 4) and the discussion section (page 45) |
| Minor |  |
| 1. Rectify the presentation error in lines 339-40, ensuring consistency in quotation formatting. | Thank you we have amended the presentation error noted. |
| 2. Attend to the typographical issues in lines 626-30. | The typography has been amended. |
| 3. Remove repetitions in lines 1002-3. | Repetition has been removed. |
| 4. Evaluate potential areas in the text where the content could be made more concise. | The paper has been proof read and amended made to sentence structure where suggested. |
| 5. Consider incorporating insights from the suggested papers on citizen social sciences. | Thank you for the inclusion of these references, they have not only enhanced this paper but will inform our work going forward. The references have been used to improve both the introduction and discussion sections. |
| Reviewer 2 |  |
| A transition sentence is needed to introduce the concept of participant experience on line 81. A bit of theoretical framing on what is meant by "participant" and "experience" would be helpful. | We appreciated this comment as the added transition statement has added clarity and improved the flow of the writing. A transition statement was added on page 5. |
| You write: "The study method must include a qualitative review of the participants' citizen science experience, either by the citizen scientists themselves or researchers." Can you explain why this was a inclusion criteria? At least some type of justification for why quantitative studies would not be included would be helpful. | A statement has been added to the methods section page 7 to justify why a qualitative approach was taken.  The paper was synthesising the qualitative experiences of participants taking part in research. Due to the nature of the synthesis quantitative data could not be incorporated in a meaningful way.  The nature of the research question to understand the experiences of young citizen scientists led us to take a qualitative approach, experience is a complex phenomena, qualitative methods allow for the exploration of the depth of human experiences which may not have been fully captured by quantitative research. The different perspectives, emotions and nuances in experiences would be best captured using qualitative data. Additionally, as far as possible we wanted to understand the context of the research experience, qualitative research allows for those contextual factors to be included and discussed in a way that quantitative data may not have provided. |
| Consider some copy editing before publication. For example, many sentences such as this one have awkward grammatical structures: “For the updated search papers were line-by-line coded by two researchers (MF 100%, 210 AS 50%, KF 50%).” Or this: “This study defines relationships as the connectedness and interactions between people and meanings attached to this human connection can be a positive side of participant experience (48,57)” | We have rephrased the sentences suggested. Additionally we have taken time to review the document as a whole before resubmission and amended several sentences for clarity. |
| Could you provide an intercoder reliability for the team coding? See this paper for example: <https://journals.sagepub.com/doi/10.1177/1609406919899220> | Thank you for this comment, we do see the relevance of intercoder reliability to foster reflexivity and dialogue in the research team. We have amended the methods to highlight that we took a reflexive process.  In line with Smith and McGannon (2018) we see it as ineffective in ensuring reliable coding as inter-rater reliability will inevitably be influenced by people’s backgrounds and theoretical stances. Additionally, our team was made up of Phd students, research assistants and professors which will have however subconsciously influenced inter coder agreement.  <https://doi.org/10.1080/1750984X.2017.1317357> |
| Do you have a reference for this statement or are you hypothesizing that this is how the system works? “As citizen scientists developed skills, knowledge and confidence and emotional understanding, their ability to communicate and interact within relationships changed, which in turn affected the power they felt and were able to use, demonstrated through decision-making.” | We have reworded the statement, it was our observations based on the literature discussed.  The literature discussed suggests that as citizen scientists acquire skills, knowledge, confidence, and emotional understanding, their capacity to communicate and interact within relationships undergoes a transformation. This, in turn, influences the power they perceive and wield, as evidenced by their decision-making. |
| On line 478 you wrote “Positive relationships with the facilitators and peer researchers often supported each other and led to positive relationships with the other stakeholders.” What about the negative relationships that were described a few paragraphs earlier – how did those change the nature of the experience? | Thank you for this interesting question. Revisiting the data there wasn’t a clear sense of how the negative relationships impacted on other elements of the experience, including other stakeholders. |
| The communication theme seems very weak and in need of further detail and justification to include in the argument. | Thank you for bringing this to our attention, we agreed on reflection that this section did not reflect our thinking or strength of the theme. We have revisited the data and strengthened this element. We believe it has greatly improved this section and appreciate your comment. (Page 26) |
| The section entitled “wider impacts” seems like a catch all of miscellaneous topics that is difficult to interpret. I suggest either deleting that from the manuscript or trying to recode into some of the other interrelated categories. | Thank you for drawing our attention to this section. We have rephrased the introductory sentence as on reflection we can see how the delineation of this section was not clear. Through our coding and discussions the literature suggested that the experiences the participants had went beyond the confines of the citizen science project, in some papers self advocacy improved within the study but in others it was clear that this continued for the participants after the study or outside of the research remit. We wanted to express this in our diagram and discussion. We agree that the codes here could be recoded into for example personal growth or relationships but we wanted to convey how this did not only occur within the research (Page 35). |
| I don’t think this statement is accurate: "As with power, relationships have not been highlighted or synthesised as an element 872 of the citizen science experience." Phillips et al (2019) describes this in adults and I believe there is a paper on Monarch Watch which describes this in youth. | Thank you for bringing this to our attention, we did not want to mislead the reader, our sentence was to convey to lack of synthesised data on young citizen scientists relationships. However, to remove any confusion we have deleted the sentence. |
| A whole paragraph is repeated (lines 1016 – 1021). | Apologies for this oversight the duplicate paragraph has been removed. |
| Figure 1 was so blurry, I could not read it. In fact, all of the figures were quite blurry | I have resubmitted the figures to PLOS one and have asked in the covering letter for their quality to be rechecked |
| In terms of the Venn diagram in Figure 2, could you provide some analytical evidence for these interrelationships beyond what is in the narrative. For example, could you provide a table showing the associations between how often communication came up between relationships and personal growth. I know it's subjective, but by putting a figure that delineates these interrelated relationships, I would want to know how strong that relationship is. Was it mentioned by one study or multiple? | By including our code book there is more clarity on how the themes were created. We see the value in this transparency.  I have additionally included a tally of how many specific codes made up a theme. However as we took an inductive approach to translating and interpreting the codes, including going back to the context of the papers we wouldn't be able to give a figure to the strength to the relationship between the overlapping themes. It was our understanding of where the themes crossed, and describing what we thought that was defined as rather than it being explicit in the text so that it could be quantified. Codes were often placed in multiple themes. The diagram is an illustration of how we saw the themes of participant experience interweaving with each other. |
| I appreciate all of the supporting documents but what I didn’t see was a codebook – I think this would be the most critical supplementary information to be able to replicate such a study. Could you please provide the full codebook you used to define and categorize your themes? | We have included a code book to aid transparency  We took an inductive approach to the synthesis with researchers independently coding and discussing their themes and codes over several sessions. The included code book is the most accurate reflection of the final coding discussed. |
