# Supplementary material for: Exploring children and young people’s experience of participating in citizen science–A qualitative evidence synthesis
Source: PLoS One. 2024 Jul 11;19(7):e0304976. doi: 10.1371/journal.pone.0304976 (PMC11239061; doi:10.1371/journal.pone.0304976)
Supplement: Attachment — Submitted filename: Response to reviewers.docx [file pone.0304976.s009.docx]

Reviewer #1: Dear Authors,

**The integration of the reviewers' comments into the revised manuscript is commendable. The approach and methodology employed in the study are robust, aligning well with standard practices within the field. However, there are areas, in particular the discussion, in the manuscript that warrant further refinement to fully realize the impact of this research.**

We appreciate you taking the time to review the paper and for commenting on our robust method. We found the comments on the concluding section particularly pertinent and have reorganised and streamlined the writing so it is more impactful; the inclusion of a dedicated limitations section allows readers to assess our work, and the overall conclusion section is more succinct, which has improved the paper. Additionally, including an explanation of key elements of Figure 2 has added clarity to the figure and the findings.

1. Results Section:
 **- To improve the manuscript's readability and cohesiveness, I suggest integrating the subsection titled "Factors Influencing Power" directly into the main "Power" section. Applying this restructuring across other sections as well will ensure that the discussion of each dimension and its influencing factors is more interconnected and accessible to the reader.**

We appreciate the comment made. There are many ways to present and discuss the data. While we agree that combining these sections may make the work more interconnected, the synthesis was not designed to search for or specifically analyse factors influencing participant experience (e.g. project design). However, the inductive coding process identified ‘factors influencing’ as a key aspect. We were keen to keep the distinction between these aspects identified and key themes of participant experience separate, as discussed:

‘Two further aspects connected to this core experience of the participants were identified: ‘ factors that influenced the experience’ and ‘ wider impact of the experience beyond the research’. These themes did not necessarily constitute participant experience, but were identified, through inductive analysis, as key aspects that impacted or were impacted by participant experience.’ Line 322

**Figure 2: While the themes in the figure are clear, the selection of factors influencing experience and the broader impacts outlined requires further clarification. As this figure is in the beginning of the results section, its basis of selection remains unclear. Addressing selection of most relevant aspects and potential omissions, especially in the "factors influencing experience" category, and elucidating on the somewhat ambiguous term "wider context" would greatly enhance the reader's understanding. The discussion section would be an appropriate place for adressing this.**

We appreciate that these sections would benefit from being specifically addressed. Thank you for suggesting these improvements. Within the findings section, further details have been added to clarify the ‘factors influencing the participant experience’ and ‘wider impacts’ aspects, specifically ‘wider context’.

Whilst focusing on one theme, it is important to situate it in the context of the wider findings, including factors influencing and wider impacts, to understand the complex tapestry fully. The ‘factors influencing participant experience’ and the ‘wider impacts’ are discussed below. As an overview, we categorised the factors influencing participant experience as a) the background and circumstance of young people, b) project design and c) wider context; see Figure 2. The background and circumstance of young people encapsulates their knowledge and skills alongside personal characteristics such as gender and ethnicity. Project design factors included space for reflection, accessibility, and time management. Wider context encompasses factors such as COVID-19, political tensions, community support, access to technology, etc., within the external environment in which the research projects operate.

The wider impacts of participant experience refer to aspects of the experience that could stay with the citizen scientist after the research project and affect the broader community. The key elements of wider impacts, highlighted in Figure 2, include personal development, improved relationships, self-efficacy and self-advocacy, and critical consciousness.

2. Discussion Section:

 **- The current discussion of the results, while comprehensive, could benefit from a more focused and sharpened narrative. I recommend a more detailed exploration of the aspects that are particularly relevant to young participants in citizen science. Specifically, it could be beneficial to:
 - Contrast the findings with existing studies that discuss influencing factors irrespective of participant age, and/or - Highlight elements uniquely pertinent to younger individuals, such as the impact of age restrictions or research topics that resonate more strongly with this demographic.**

Thank you for acknowledging that our discussion section was comprehensive. We have made some adjustments to the discussion. Line 913 now raises an issue of particular importance to working with young people.

One area that may be of specific relevance to young people was the idea that citizen scientists began to see the system as weighted against them (53). Makuch (68) suggests citizen science can empower children to contribute to local and global issues. However, at such a key stage of development, if projects are not defined with clear outcomes, they can cause disillusionment for the young citizen scientists (53, 78).

By emphasising the general literature in the sentence below, the contrast with the findings of the young people’s experience here and in the remainder of the paragraph are emphasised.

Relationships in the general citizen science literature often refer to the relationship with science rather than the relationship between peers, researchers and others involved in the project, which are key to the child's experience (68). Relational empowerment is key to children increasing their networks or ‘bridging worlds’, which can bridge social divisions and improve children's ability to collaborate (69). Planning space and time for these relationships to develop should be integral to citizen science projects involving young people to improve their experience.

We have also moved content related to stakeholder relationships and individual experience to the discussion to streamline the conclusion.

Skarlatidou (84) evidences the stakeholders involved in citizen science. For example, civil society organisations, government agencies and academic organisations. Understanding the communication between these stakeholders and young participants is an interesting area of future research.

and

The participant experience was broken down and discussed as separate themes throughout the findings. However, Wagaman’s (61) paper describes part of the participant experience, which was important to the citizen scientist, feeling connected to a researcher who she thought was transgender like herself. When the citizen scientist and the researcher met, the citizen scientist discovered she was a cisgender female. This specific experience of feeling connected to the facilitator because of her profile and then having to readjust when they met, in reality, does not fit easily into a specific theme. Yet, it is still a part of this young citizen scientist experience. This example emphasises how context-specific these experiences can be and suggests that the elements of participant experience are unique to each young participant in each research study.

**- Wider Impacts Subsection: The purpose of this subsection seems to be primarily to advocate for standardized evaluations in citizen science projects. While important, this could be succinctly addressed in the conclusions. Instead, a justification for the chosen aspects of wider participation as depicted in Figure 2 would be more beneficial in this context.**

Thank you for drawing our attention to this section. We agree that the amended content has improved the paper. The section has been rewritten, and the paragraph regarding evaluations has been condensed and moved to the concluding section for future research. We have related the aspects in wider impacts to the wider literature to give this section a clearer purpose.

This study identified key wider impacts such as personal development, improved relationships, self-efficacy, self-advocacy, and critical consciousness. Personal development included elements such as educational, social, and personal growth of the young citizen scientists that could remain after the project. The wider literature discusses increasing scientific literacy (73, 68) alongside the potential for self-efficacy and self-advocacy through participation in citizen science (70, 78). However, more evidence is needed to support sustained change beyond the project in young people, highlighting an area for future research.

Within the findings, critical consciousness was said to give youth an understanding of injustice (35,38,53). However, as discussed within the power theme, if the citizen scientists research was not acted on, the participants were disheartened by the lack of change (53). Khan’s (2022) paper highlights the need to move beyond critical consciousness to enable child-led knowledge, collaboration, and support to challenge ‘the white institutional worlds’ by which young people are governed rather than simply understanding and raising awareness (78). Future projects must understand and plan for a positive participant experience, specifically when including multicultural youth, to ensure that young people are not burdened by cultural labour.

**- Concentration on Limitations**: The "Strengths and Limitations" section should be streamlined to emphasize the limitations of the methodological approach. This focus will provide readers with a clearer understanding of the study's scope and the reliability of its findings.**

Thank you for this comment; we appreciate the need to identify limitations in academic articles. We have concentrated the focus of this section on limitations and retitled the subsections.

Limitations

A key limitation of the synthesis is that although all studies included met the eligibility of being ‘citizen science’ projects, several methodological approaches were included, such as YPAR and community research. The drawback to this is that the research team has classified the study as citizen science rather than seeking the opinion of the authors of the studies - the study hadn't been designed from the start with characteristics and principles of citizen science in mind (10,22). Although participant experience was discussed in each of the papers selected, the aim of the study was not always to evaluate participant experience, which is a key citizen science principle (10). The methods used within the synthesised studies and the quality of analysis also varied.

The search terms for this synthesis included children and young people (Mean age under 25). The 33 included papers consisted of studies with citizen scientists aged 9 and upwards, mainly teenagers and young adults. The experience of younger children taking part in a research project will be very different from that of someone in their early 20s, and this needs to be explored.

Despite the common themes identified, for each young citizen scientist, the experience was unique and situated within a specific context. While beneficial for synthesising complex information, thematic analysis faces limitations in capturing themes' interconnected and independent nature, making it difficult to convey these relationships effectively. Although this is represented in Figure 2, it is difficult to express this through written narrative. The impact of any single factor will be related to and dependent on other factors, motivations, context, and the individual.

The research followed a robust methodology a systematic approach to identifying papers, followed by independent screening and critical appraisal by a team of reviewers. Nevertheless, it is important to acknowledge that qualitative synthesis invites subjectivity and is interpretive in nature. To ensure the credibility of our synthesised themes, we continuously referred back to the original articles and took a collaborative approach to reach conclusions.

**- Conclusive Summary: A concluding section that precedes or is integrated with "Future Suggestions" appears necessary to succinctly summarize the key messages derived from the analysis of youth experiences in citizen science. Highlighting some of the strengths mentioned previously, along with a compelling articulation of how young people represent a distinct group with specific needs and impacts within citizen science, will lay a solid groundwork for future research.**

The strengths and future research suggestions have been combined, and a concluding section has been added. Additionally, this section has been streamlined.

Strengths of the project and future suggestions.

This synthesis offers researchers a comprehensive understanding of the experiences of young citizen scientists. Examining various studies collectively provides a more inclusive perspective on these experiences. For instance, it combines the experiences of indigenous groups, youth of colour, transgender individuals, and studies involving both single and mixed genders. This approach aims to uncover the common experiences shared by young people engaged in citizen science activities.

A key principle of citizen science is that citizen scientists and researchers benefit from participating (10). Future research could compare the participant experience within specifically designed citizen science projects with the experience of participants from different participatory approaches. Additionally, the studies synthesised fall under different typologies of citizen science studies (82,83) and different disciplines. Creating a synthesis that considers the various types of citizen science and if they are from the social or natural sciences could allow for further comparisons and new insights into young people’s experiences.

Numerous citizen science studies were not included in this synthesis as participant experience was not reviewed or evaluated within them. Some studies within the synthesis do not have participant experience as a primary aim. This urgently needs to be addressed to ensure young people benefit from giving their time to create new knowledge through research.

Key conclusions

This research is the first qualitative evidence synthesis, to our knowledge, that has reviewed and thematically analysed qualitative research discussing the participant experience of young citizen scientists. 33 papers were reviewed. The key takeaways are the themes of power, relationships and personal growth interlinked by communication, self-efficacy and decision-making. It stresses the importance of including children and young people in research that impacts them without causing harm. By focusing on the distinct needs and impacts of young people in citizen science, this study sets a foundation for future research to enable projects beneficial for young participants.

Minor Issues:

**Table 1 Presentation: The right side of Table 1 is truncated, which may hinder the comprehension of the data presented. A review and adjustment of the table's layout are recommended.**

The table has been submitted in line with PLOS one’s guidance

- Size.
  Tables do not have strict width and height requirements. Do not split your table or otherwise try to make the table appear within the manuscript margins if it does not fit on one page. In Word, tables that run off of the manuscript page can be seen using Draft View. In the PDF version of the published article, very wide tables may be printed sideways, and long tables may span more than one page.

We are happy to work with the publication to address any issues with the table presentation.

**Legibility of Figures: Figures 1 and 2 suffer from legibility issues, potentially due to the PDF export process. Ensuring the clarity and readability of these figures is crucial for readers to fully grasp the presented data and analyses.**

We are happy to work with PLOSONE on the quality of the figures. The figures are of high quality, produced in CANVA, downloaded in the correct format to submit to PACE, and then uploaded to the system. We agree that the quality when downloaded through the PLOSONE portal is not acceptable and are happy to work with the team to improve the appearance of the figures.

**By addressing these issues, I am confident that your article will significantly influence further research on this topic.**

Thank you again for your input into our paper and for this comment, which encourages us to improve the paper so that our research can be published.

**Reviewer #2: Thank you for addressing each of the reviewer's comments. I am satisfied with the diligence the authors took to improve the paper.**

Thank you for your original comments on the paper and for reviewing our improvements. We appreciate your time and effort in suggesting the original improvements.
