# Supplementary material for: Exploring children and young people’s experience of participating in citizen science–A qualitative evidence synthesis
Source: PLoS One. 2024 Jul 11;19(7):e0304976. doi: 10.1371/journal.pone.0304976 (PMC11239061; doi:10.1371/journal.pone.0304976)
Supplement: Attachment — Submitted filename: reviewer response .docx [file pone.0304976.s010.docx]

Dear Dr Cloete

Thank you, Dr Cloete, for your quick response and for providing feedback on our manuscript.

We are pleased that our responses to the reviewers' comments were deemed commendable. We understand the importance of ensuring clarity and accuracy in our manuscript, and it has been thoroughly proofread, with changes visible in the track changed manuscript. We appreciate the reminder to adhere to PLOS ONES referencing style and have amended the in-text citations.

We are committed to meeting the journal requirements. As such, we have double-checked the reference list to ensure it is complete and correct and made some modifications to ensure it conforms to PLOS ONE's requirements. Sight corrections were made to references 2, 6, 7, 10, 38, and 56. This is evidenced in the tracked changes document. We apologise that these references may not have met PLOS ONE's requirements.

Again, Thank you for your valuable feedback and for considering our manuscript for publication in PLOS ONE.

Sincerely,

Marie Frazer
